# A Multimodal Data Fusion and Embedding Attention Mechanism-Based Method for Eggplant Disease Detection

**DOI:** 10.3390/plants14050786

**Published:** 2025-03-04

**Authors:** Xinyue Wang, Fengyi Yan, Bo Li, Boda Yu, Xingyu Zhou, Xuechun Tang, Tongyue Jia, Chunli Lv

**Affiliations:** 1 China Agricultural University, Beijing 100083, China; 2 College of Mathematics and Physics, North China Electric Power University, Beijing 102206, China; 3 School of Economics and Management, Beijing Forestry University, Beijing 100083, China; 4 School of Foreign Languages, Beihang University, Beijing 100191, China

**Keywords:** artificial intelligence in agriculture, eggplant disease detection, deep learning, multimodal data fusion

## Abstract

A novel eggplant disease detection method based on multimodal data fusion and attention mechanisms is proposed in this study, aimed at improving both the accuracy and robustness of disease detection. The method integrates image and sensor data, optimizing the fusion of multimodal features through an embedded attention mechanism, which enhances the model’s ability to focus on disease-related features. Experimental results demonstrate that the proposed method excels across various evaluation metrics, achieving a precision of 0.94, recall of 0.90, accuracy of 0.92, and mAP@75 of 0.91, indicating excellent classification accuracy and object localization capability. Further experiments, through ablation studies, evaluated the impact of different attention mechanisms and loss functions on model performance, all of which showed superior performance for the proposed approach. The multimodal data fusion combined with the embedded attention mechanism effectively enhances the accuracy and robustness of the eggplant disease detection model, making it highly suitable for complex disease identification tasks and demonstrating significant potential for widespread application.

## 1. Introduction

Eggplant, as an important economic crop, is widely cultivated in various regions worldwide [1,2,3]. It holds significant economic and nutritional value in agricultural production; however, its cultivation process is often threatened by various diseases, such as gray mold, wilt, and anthracnose [4,5]. These diseases not only directly cause yield and quality reductions but may also lead to environmental pollution due to excessive pesticide use, posing a severe challenge to the sustainable development of agriculture. Moreover, early detection and precise control of diseases are crucial for reducing economic losses and improving the effectiveness of eggplant cultivation [6,7].

Traditional disease detection methods primarily rely on manual observation and expert judgment [8]. Farmers or agricultural technicians typically identify diseases by visually inspecting symptoms of lesions on eggplant leaves, stems, and other parts. While this method is somewhat practical, its limitations are evident. First, manual detection is inefficient, especially in large-scale planting scenarios, where rapid and comprehensive disease monitoring is difficult. Second, the accuracy of manual identification is highly dependent on the expertise of the inspector, leading to significant subjective differences that can affect diagnostic accuracy. Furthermore, for some early-stage diseases or those with inconspicuous symptoms, manual detection may fail to identify the problem in a timely manner, missing the optimal intervention window [9].

In recent years, with the rapid development of deep learning technologies, significant progress has been made in disease detection methods based on deep learning [10,11,12]. These methods construct deep neural networks to automatically extract features from large datasets, effectively overcoming the shortcomings of traditional approaches. For instance, convolutional neural networks (CNNs) are widely used in agricultural disease detection, as they can extract fine-grained features of diseased regions from image data, achieving high classification accuracy [13]. Iftikhar et al. [14] explored a deep learning-based disease detection method, specifically utilizing a CNN architecture for automatic plant disease detection. Their study focused on three different crops (apple, maize, and potato), providing an in-depth performance analysis of hyperparameters within the context of plant disease detection. Additionally, they analyzed the impact of data augmentation. Experimental results show that their fine-tuned enhanced CNN model (E-CNN) achieved an accuracy of 98.17% for fungal diseases. Thakur et al. [15] introduced a lightweight CNN, ‘VGG-ICNN’, for recognizing crop diseases using plant leaf images. Experimental results demonstrate that this method outperformed several recent deep learning approaches, achieving an accuracy of 99.16% on the PlantVillage dataset.

Additionally, object detection algorithms such as RetinaNet and YOLO are increasingly being applied to disease region localization and detection tasks. These models not only improve detection efficiency but also provide more precise boundaries for disease-affected areas. Sunitha et al. [16] combined the RetinaNet object detection architecture with ViT, proposing a two-stage detector system to improve wheat head detection performance. Experimental results on the wheat head detection dataset indicate that the proposed RetinaNet+ViT model is an effective method for wheat head detection. Kumar et al. [17] proposed and evaluated a precise method for detecting rice leaf diseases, using the DenseNet-Bi-FAPNwithYOLOv5 model, which integrates the YOLOv5 network with DAIS segmentation and Bi-FAPN networks. Experimental results demonstrate that their method achieved an accuracy of 94.87%, aiding farmers in early-stage rice leaf disease detection. To address small object detection challenges, Sangaiah et al. [18] proposed a drone-based T-YOLO-Rice network for rice leaf disease detection. The network architecture was modified based on T-YOLO v4 by adding extra YOLO layers, other modules, and additional convolutional layers. This network outperformed other machine learning architectures, also providing onboard intelligence and small object detection.

However, existing deep learning methods still face several challenges in practical applications. Firstly, most models heavily rely on large amounts of labeled data, which is often lacking in the agricultural domain, particularly in the detection of rare diseases. Secondly, many existing methods focus on processing single-modality data, making it difficult to leverage multimodal data (such as image and text) comprehensively, thus limiting the overall performance of the models. Additionally, the computational complexity of these models is high, making it challenging to deploy them efficiently on resource-constrained edge devices. To address these issues, this study proposes a disease detection and control method for eggplant based on multimodal data and attention mechanisms, aiming to overcome the bottlenecks in data utilization, model performance, and practical deployment. The main innovations of this study are as follows:

- Multimodal Data Fusion: By introducing a novel multimodal fusion embedding module, image data and sensor data are effectively combined to utilize multisource information. This approach directly integrates environmental data from sensors with visual data from images, which significantly enhances disease detection accuracy and robustness, especially under varying environmental conditions.
- Embedded Attention Mechanism: A new embedded attention mechanism is designed to enhance the learning of important disease-related features while suppressing irrelevant or redundant information that may interfere with detection results. Unlike traditional attention mechanisms that focus only on image features, our mechanism simultaneously optimizes both image and sensor data, ensuring the model focuses on the most relevant regions and conditions that lead to disease outbreaks. This approach significantly improves the model’s ability to detect diseases in complex environments, which is a major advancement over conventional techniques.
- Embedded Loss Function: A novel embedded loss function is proposed to optimize the joint representation learning of multimodal data (image and sensor data), further improving the model’s training efficiency and convergence performance. This loss function is specifically designed to work with the multimodal inputs, ensuring better alignment between the image and sensor data during the training process, thereby enhancing overall detection performance.

In conclusion, by integrating multimodal data with attention mechanisms, this study proposes an innovative method for disease detection and control. This method not only effectively addresses the limitations of traditional approaches but also provides new technological solutions and application references for agricultural disease management.

## 2. Related Work

### 2.1. Multimodal Data

In the field of agricultural disease detection, the introduction of multimodal data has gradually become an important means of improving detection accuracy and generalization capabilities [19,20]. Multimodal data refers to data originating from different sources or types, such as image data, text descriptions, sensor information, etc., which can represent the same object or event from multiple perspectives [21,22]. The theoretical foundation for multimodal data fusion lies in the joint modeling of feature representations. Let the feature representation of image modality data be $F_{v}\in R^{H\times W\times C}$, where *H* and *W* represent the height and width of the feature map, respectively, and *C* represents the number of channels; the embedded feature representation of textual modality data is $F_{t}\in R^{D}$, where *D* is the dimension of the embedding vector. To fuse these two modalities of data, feature alignment and mapping are necessary to place them in the same feature space. The fused joint feature $F_{m}$ can be represented as

(1) $$F_{m}=f_{\mathrm{fusion}}\left(F_{v},F_{t}\right)$$

where $f_{\mathrm{fusion}}$ is the multimodal data fusion function, which can be implemented in various forms. Common fusion methods include weighted linear fusion, concatenation operations, and attention mechanisms. Weighted linear fusion is a simple and effective multimodal fusion strategy. Concatenation is another common fusion method, where the image and text features are directly concatenated into a high-dimensional representation:

(2) $$F_{m}=\mathrm{Concat}\left(F_{v},F_{t}\right)$$

where Concat denotes the concatenation operation. Although this method is simple and straightforward, the resulting high-dimensional features may significantly increase computational costs. To improve the efficiency and effectiveness of feature fusion, modern studies often introduce attention mechanisms. Attention mechanisms weight the multimodal features to highlight key information and suppress redundant features. Given image features $F_{v}$ and text features $F_{t}$, the weighted joint representation can be expressed as

(3) $$F_{m}=\mathrm{Softmax}\left(\frac{{\mathrm{QK}}^{⊤}}{\sqrt{d}}\right)V$$

where $Q=W_{q}F_{m}$, $K=W_{k}F_{m}$, and $V=W_{v}F_{m}$ represent the query, key, and value matrices, respectively; $W_{q}$, $W_{k}$, and $W_{v}$ are learnable weight matrices, and *d* is the scaling factor for the feature dimension used to normalize the attention scores. The advantage of multimodal data lies in its ability to integrate complementary information from different modalities. For instance, in disease detection tasks, image data can provide intuitive visual information, such as the shape, color, and texture of lesions, while textual data can provide background knowledge, such as conditions for disease occurrence, symptom descriptions, and historical data. This complementarity allows multimodal methods to demonstrate higher detection accuracy and robustness when dealing with complex scenarios, such as the coexistence of multiple diseases or rare diseases [23,24]. Furthermore, multimodal fusion can significantly enhance a model’s generalization ability, enabling it to provide reliable detection results even when the quality of one modality’s data is low or missing.

Despite the immense potential of multimodal data in agricultural disease detection, its application still faces several challenges. Firstly, the heterogeneity of multimodal data is a core issue. Different modalities exhibit significant differences in feature representation distributions, and direct fusion may introduce noise or lead to information loss. To quantify this difference, the Kullback–Leibler divergence ($D_{\mathrm{KL}}$) can be used to measure the deviation between the feature distributions of image data $P(F_{v})$ and text data $P(F_{t})$:

(4) $$D_{\mathrm{KL}}\left(P\left(F_{v}\right)\|P\left(F_{t}\right)\right)=\int P\left(F_{v}\right)\log\frac{P(F_{v})}{P(F_{t})}dF$$

Minimizing this distributional difference is crucial for efficient feature alignment. Secondly, the high-dimensional features resulting from multimodal fusion can lead to a sharp increase in computational complexity. Assuming the feature dimension is *d*, the computational complexity of fusion typically scales as $O(d^{2})$, which poses a significant challenge in resource-constrained agricultural scenarios (e.g., edge device deployment) [23,25]. As a result, researchers have attempted to introduce lightweight models and distributed computing strategies to reduce computational costs. Finally, the acquisition and annotation costs of multimodal data are relatively high. Data in the agricultural field is often scattered across fields, and data collection conditions can be complex, especially when high-quality text descriptions require domain expertise [26,27]. Potential solutions to this problem include utilizing transfer learning, semi-supervised learning, and GANs to generate multimodal synthetic data, which could alleviate the issue of insufficient data.

### 2.2. Attention Mechanism

The attention mechanism is a technique aimed at improving model performance by focusing on the most important information. Initially proposed for natural language processing tasks, it has since been widely applied to image processing, time series analysis, and multimodal tasks [28,29,30]. Its theoretical foundation is rooted in selective attention in human cognition, which prioritizes key information while disregarding less relevant or redundant data. In deep learning, the attention mechanism dynamically allocates weights and selectively extracts the most relevant parts from input features, enabling excellent performance when handling complex, heterogeneous data.

The mathematical principle of the attention mechanism can be described as a weighted sum process over queries (query), keys (key), and values (value), with the weights computed based on the similarity between the query and key [31]. In agricultural disease detection tasks, the attention mechanism has been widely used to improve detection accuracy and generalization capabilities [32,33]. For example, CNNs may overlook fine-grained disease features due to their fixed receptive field sizes when processing large-scale image data. By introducing attention mechanisms, models can adaptively focus on disease regions, enhancing the ability to capture affected areas [34,35].

A typical example is the channel attention mechanism, which enhances key features by calculating the importance weights for each feature channel. Given a feature map $F\in R^{H\times W\times C}$, global average pooling (GAP) is first performed along the spatial dimensions to obtain the channel weights $z\in R^{C}$:

(5) $$z_{c}=\frac{1}{H\times W}\sum_{i=1}^{H}\sum_{j=1}^{W}F_{i,j,c}$$

Then, a two-layer fully connected network generates weight scores $s\in R^{C}$, which are multiplied by the original features per channel:

(6) $$F_{c}^{\prime }=s_{c}\cdot F_{c}$$

This method effectively highlights important channels, thereby improving the model’s sensitivity to disease features. Additionally, the spatial attention mechanism focuses on the spatial information of images by assigning weights to different spatial locations, highlighting areas where diseases are present [34,36]. The calculation typically involves pooling operations (such as max pooling or average pooling) along the channel dimension, producing a two-dimensional feature map $F_{s}\in R^{H\times W}$, followed by convolutional layers to generate attention weights:

(7) $$F_{s}^{\prime }=\sigma \left(\mathrm{Conv}2D\left(\left[\mathrm{AvgPool}\left(F\right),\mathrm{MaxPool}\left(F\right)\right]\right)\right)$$

Here, σ represents the activation function (e.g., Sigmoid), and Conv2D denotes the two-dimensional convolution operation. Different types of attention mechanisms have distinct advantages in agricultural disease detection tasks. Channel attention is suitable for improving the representation of global information, while spatial attention is more adept at capturing local disease features. Self-attention mechanisms, on the other hand, perform particularly well in handling complex multitarget associations in diverse scenarios [37]. However, these methods also present certain challenges. For instance, the computational complexity of attention mechanisms, especially self-attention, increases quadratically with the size of the feature map:

(8) $$O(n^{2}\cdot d)$$

where *n* is the spatial resolution of the feature map, and *d* is the feature dimension. To mitigate this complexity, various improvements, such as sparse attention and factorized attention, have been proposed to reduce computational overhead [38].

### 2.3. Knowledge Graphs and Expert Systems

The application of knowledge graphs and expert systems in agricultural disease control has gradually become an important research direction in intelligent agriculture. Knowledge graphs are a technique for structurally representing knowledge in the form of graphs, where nodes represent entities (e.g., disease types, symptoms, environmental conditions), and edges represent relationships between entities. By constructing knowledge graphs related to agricultural diseases, heterogeneous data from multiple sources can be effectively integrated, providing comprehensive knowledge support for disease detection and prevention [39]. Expert systems, on the other hand, are computer systems based on rule-based reasoning, with a core consisting of a knowledge base and a reasoning engine that can deduce diagnostic results or action recommendations based on input information [40]. When combined, knowledge graphs and expert systems can significantly enhance the system’s intelligence and decision-making capabilities.

The construction of knowledge graphs relies on the extraction, representation, and storage of knowledge [41]. Let $E=e_{1},e_{2},\ldots ,e_{n}$ represent a set of entities and $R=r_{1},r_{2},\ldots ,r_{m}$ represent a set of relationships, the knowledge graph can be represented as a collection of triples $G=\left(e_{i},r_{k},e_{j}\right)|e_{i},e_{j}\in E,r_{k}\in R$, where each triple $\left(e_{i},r_{k},e_{j}\right)$ indicates that the entities $e_{i}$ and $e_{j}$ are connected through the relationship $r_{k}$. For example, the relationship between “eggplant” and “gray mold” can be represented as (eggplant, susceptible to, gray mold).

To facilitate computer processing and reasoning, entities and relationships in the knowledge graph are typically embedded in a low-dimensional vector space. Let the embedding of entity $e_{i}$ be $e_{i}\in R^{d}$ and the embedding of relation $r_{k}$ be $r_{k}\in R^{d}$, the validity of the triple $\left(e_{i},r_{k},e_{j}\right)$ can be measured by a scoring function $f(e_{i},r_{k},e_{j})$. For example, the scoring function based on the TransE model is

(9) $$f\left(e_{i},r_{k},e_{j}\right)=-{\left|e_{i}+r_{k}-e_{j}\right|}_{2}$$

where ${\left|\cdot \right|}_{2}$ denotes the Euclidean norm. By optimizing the scoring function, low-dimensional embeddings of entities and relations can be learned. The application of knowledge graphs in agricultural disease control is mainly reflected in two aspects. First, they can provide background knowledge related to diseases. For instance, the occurrence of certain diseases may be closely linked to specific environmental conditions (e.g., humidity, temperature). Knowledge graphs can explicitly represent this information, assisting the system in inferring the possible causes of diseases [42]. Second, knowledge graphs support the fusion of cross-modal information. By associating image data, textual descriptions, and other multimodal information with semantic information from the knowledge graph, deeper knowledge reasoning can be achieved. For example, when an image identifies black spots on leaves, the knowledge graph can provide a list of disease candidates related to black spots, narrowing down the diagnostic scope [43].

In the integration of multimodal data and knowledge graphs, the key challenge is how to align multimodal features with knowledge representations [44]. Let the feature of the image modality be $F_{v}\in R^{d}$, the embedding of the text modality be $F_{t}\in R^{d}$, and the embedding of an entity in the knowledge graph be $e\in R^{d}$. The alignment of multimodal features and knowledge embeddings can be represented by a matching function g(·):

(10) $$g\left(F_{v},e\right)=\cos\left(F_{v},e\right),\;g\left(F_{t},e\right)=\cos\left(F_{t},e\right)$$

where cos(·,·) represents the cosine similarity. By maximizing the matching score, multimodal features can be aligned with the knowledge graph for improved task performance.

## 3. Materials and Methods

### 3.1. Dataset Collection

**Figure 1:**
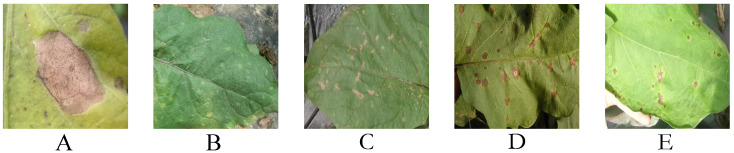
Dataset samples: (**A**) Fusarium wilt. (**B**) Leaf mold. (**C**) Bacterial leaf spot. (**D**) Brown stripe. (**E**) Brown spot.

In this study, the dataset collection is one of the key steps in eggplant disease detection. To build a representative and diverse dataset for disease images, images related to eggplant diseases were collected from Bayan Nur City, Inner Mongolia, and online image resources, spanning from March 2023 to October 2024. The collected disease types include brown spot disease, brown stripe disease, fusarium wilt, leaf mold, and bacterial leaf spot, as shown in Figure 1. As summarized in Table 1, the number of images for each disease ranges from 700 to 1800, ensuring the diversity and sufficient sample size of the dataset for effective learning and analysis of different diseases. The primary sources of data collection include field collections from eggplant planting bases in Bayan Nur City and publicly available online data [45]. For the field collection, collaboration with local farms allowed for the acquisition of eggplant samples at different growth stages. During the collection, high-resolution digital cameras (such as Canon EOS 5D with an 18–55 mm lens) were used to ensure the clarity and preservation of image details. The focus was specifically on capturing the disease symptoms, not the entire plant, ensuring that the images focus on the affected areas for accurate disease detection. The images were taken from a distance of 30–50 cm to capture detailed symptoms while avoiding distortion. Artificial illumination was not used, and all images were taken under natural lighting conditions to simulate realistic farm environments.

During the collection process, particular attention was paid to the different manifestations of the diseases, such as the color changes in brown spot disease lesions, the texture changes in brown stripe disease, the extent of fusarium wilt lesions, and the distribution of mold in leaf mold. These characteristics are often closely related to environmental factors; therefore, different time periods were selected for image capture under different weather conditions to capture the changes in the diseases under varying environmental circumstances. Additionally, to ensure the dataset’s comprehensiveness, images were collected from different parts of the eggplant plant (such as leaves, stems, and fruits) to ensure that the model could recognize disease manifestations in various parts. For each disease, samples were selected based on their prevalence and pathogenicity in the field. For example, brown spot disease and brown stripe disease are more common in the eggplant planting areas of Bayan Nur City. These diseases often manifest as irregular brown spots or streaks on the leaves, typically accompanied by yellowing and wilting. Brown spot disease usually presents larger spots with clear borders, while brown stripe disease shows long, striped lesions with varying color shades, often dark brown or gray-brown. Fusarium wilt, caused by fungi, primarily manifests as white mold on the leaves, with lesions ranging from small spots to large patches, and the mold rapidly spreads as the disease progresses. Leaf mold, another common fungal disease, shows yellow or green lesions that eventually turn white or gray, with a prominent mold layer on the leaves. Bacterial leaf spots appear as wet, round, or irregular-shaped lesions on the leaves, with blurred edges, often expanding into larger yellow spots. To further enhance the diversity of the dataset, additional images were collected from online resources, primarily from agricultural websites, disease recognition datasets from scientific papers, and public farm data. The online data provided more disease type samples and included eggplant disease images from various regions and environments, which is crucial for improving the model’s generalization ability. The online data were sourced from well-known agricultural research institutions’ publicly available image libraries to ensure the accuracy and authenticity of the data.

In addition to the image dataset, sensor data were also collected and integrated to enhance the multimodal learning capability of the eggplant disease detection model. The introduction of sensor data provides a more comprehensive understanding of the growth environment and physiological conditions of the eggplant, and when combined with image data, it contributes to improving disease detection accuracy and robustness. The collection process for sensor data is more complex, involving the configuration of multiple types of sensors, data collection methods, data preprocessing, and integration with disease detection tasks. Therefore, describing this process in detail is crucial to understanding how multimodal data are applied in disease detection. To ensure the diversity and comprehensiveness of the data, various types of sensors were selected for data collection. The sensor types mainly include environmental monitoring sensors such as temperature and humidity sensors, light sensors, and gas sensors. The temperature and humidity sensor is an important type of environmental sensor, primarily used to monitor changes in temperature and humidity in the eggplant growth environment. Plants such as eggplants are highly sensitive to changes in temperature and humidity, especially when the environment is highly humid, as certain diseases (such as leaf mold and fusarium wilt) are more likely to occur. The DHT22 temperature and humidity sensor, being a more affordable option, is well suited for this study’s purpose due to its reasonable accuracy for monitoring large-scale environmental changes. The DHT22 sensor measures temperature within a range of −40 to 80 °C, with an accuracy of ±0.5 °C, and humidity from 0% to 100%, with an accuracy of ±2–5%. Although it may not offer the high precision found in more expensive, laboratory-grade sensors, it provides sufficiently reliable data for environmental monitoring in agricultural settings. To ensure the reliability of the data, the DHT22 sensors were periodically calibrated and checked to minimize any measurement errors. Therefore, a DHT22 temperature and humidity sensor was used to collect 119,823 data points. This sensor accurately measures the environment’s temperature and humidity, transmitting the data to the data collection system, providing precise environmental data for subsequent analysis. The data collection frequency for each sensor was set to once per hour to ensure timeliness and to avoid excessive data volume.

In addition to temperature and humidity, light also plays a crucial role in plant growth, as excessive or insufficient light can hinder plant development and even affect its disease resistance. Therefore, a light-dependent resistor (LDR) was used as a light sensor to monitor the light intensity in the eggplant growth area, collecting 100,927 data points. The light sensor data were also collected every hour to capture light intensity variations that might influence disease development, as unhealthy growth conditions often correlate with weak resistance, making plants more susceptible to diseases. Additionally, a gas sensor was used to monitor the concentration of carbon dioxide (CO_2_) in the air of the eggplant cultivation area, collecting 103,955 data points. Changes in CO_2_ concentration affect plant photosynthesis, and although CO_2_ itself does not directly cause diseases, abnormal CO_2_ levels can indirectly impact plant health. Thus, CO_2_ sensor data provides valuable information for identifying potential disease risks. Each sensor collected data every hour to ensure that all environmental factors were continuously monitored without gaps. Multiple sensors of the same type were deployed across each field to ensure comprehensive data collection from various locations within the field. Specifically, each type of sensor was placed in representative locations to provide accurate and diverse environmental data. For instance, in each field, multiple temperature and humidity sensors were placed in different zones to account for possible microclimates, while light sensors were strategically placed in different parts of the cultivation area to monitor the full light variation throughout the space. The placement of these sensors ensured that the environmental data were representative of the entire growing area, rather than limited to a single pot or specific location. The data collection was conducted via wireless transmission modules that sent data from the sensors to a cloud server for storage and processing. Data transmission was carried out using wireless modules such as Zigbee and Wi-Fi to ensure stable and timely uploading to the data platform, avoiding data loss caused by network disruptions. This wireless transmission ensured that the data were continuously collected and stored without the need for manual intervention, contributing to the efficiency and accuracy of the overall system. To ensure the accuracy of data collection, all sensors were periodically calibrated and checked to ensure that each sensor’s measurements were accurate and representative.

### 3.2. Dataset Preprocessing

#### 3.2.1. Image Dataset Enhancement

In agricultural disease detection tasks, image data augmentation is a widely used technique that enhances the diversity of the dataset and improves the generalization capability of the model by applying transformations or generating new synthetic data from the original images. In particular, in the context of disease detection, where labeled data are often limited and imbalanced, data augmentation techniques can effectively mitigate the issue of scarce data. CutMix is a data augmentation method that enhances the dataset by mixing both the images and their corresponding labels. The key idea is to randomly crop a region from two images and mix the pixel values of one image into the other while adjusting the corresponding label distribution based on the proportion of the cropped region. Let the two images be $X_{A}$ and $X_{B}$, with labels $y_{A}$ and $y_{B}$, and let the ratio of the cropped region be λ. The augmented image and label can be represented as

(11) $$X=\lambda X_{A}+\left(1-\lambda \right)X_{B},\;y=\lambda y_{A}+\left(1-\lambda \right)y_{B}$$

where λ∈[0,1] is a weight randomly sampled from a Beta distribution, controlling the size of the cropped region. By mixing both pixel values and labels, CutMix not only increases the diversity of the data but also encourages the model to learn more robust feature representations. However, to ensure that the generated images still include the symptoms of the disease, we specifically make sure that the cropped regions containing the disease symptoms from both images are kept intact during the mixing process. The cropped region from each image is selected such that the area containing the disease lesion is preserved, preventing the possibility of the symptom being removed or incorrectly labeled. Mosaic, another widely used enhancement method in object detection tasks, involves stitching four images into a new image, enabling the model to learn multiple contextual information within a single input. Specifically, let the four images be $X_{1}$, $X_{2}$, $X_{3}$, and $X_{4}$, with corresponding sizes $\left(h_{i},w_{i}\right)$. By adjusting the positions and scaling of these images, they can be combined into a new image $X_{\mathrm{Mosaic}}$, which is mathematically expressed as

(12) $$X_{\mathrm{Mosaic}}\left(x,y\right)=\left\{\begin{array}{cc} X_{1}\left(x,y\right), & \mathrm{if}\;x\in \left[0,w_{1}\right],y\in \left[0,h_{1}\right]\\ X_{2}\left(x-w_{1},y\right), & \mathrm{if}\;x\in \left[w_{1},w_{1}+w_{2}\right],y\in \left[0,h_{2}\right]\\ X_{3}\left(x,y-h_{1}\right), & \mathrm{if}\;x\in \left[0,w_{3}\right],y\in \left[h_{1},h_{1}+h_{3}\right]\\ X_{4}\left(x-w_{3},y-h_{2}\right), & \mathrm{if}\;x\in \left[w_{3},w_{3}+w_{4}\right],y\in \left[h_{2},h_{2}+h_{4}\right] \end{array}\right.$$

This method, by stitching information from different images, enables the model to better adapt to multiple scales and complex scenarios, thereby improving detection performance. GridMask, a data augmentation strategy based on masking mechanisms, aims to force the model to learn more global feature representations by adding regularized occlusion areas to the image. Let the input image be X and the mask for the occlusion area be M. The augmented image is then represented as

(13) $$X^{\prime }=X⊙M$$

where ⊙ denotes element-wise multiplication, and M is a binary matrix with a grid structure that specifies the position of the occlusion areas. The size and spacing of the grid can be randomly generated to ensure diversity in data augmentation. By randomly occluding parts of the image, GridMask can effectively prevent the model from over-relying on local information, enhancing its ability to perceive global features. However, to ensure that the symptoms of the disease are still visible and included in the training, we ensure that the occlusion grid does not cover the areas where the disease symptoms are present. We take care to position the occlusion mask in regions that do not overlap with critical areas, such as lesions or mold spots. This guarantees that, even with partial occlusion, the disease symptoms remain visible and correctly labeled, preserving the integrity of the annotated disease regions.

#### 3.2.2. Text Dataset Embedding

Text data embedding is a fundamental technique in natural language processing (NLP), aimed at mapping text data into continuous low-dimensional vector spaces, enabling more efficient understanding and processing of semantic information by computers. This embedding representation method captures not only the semantic relationships between words but also the overall meaning of sentences and paragraphs at higher levels. In agricultural disease detection tasks, the multimodal fusion of text data (such as disease descriptions, symptom records, and environmental conditions) with image data can significantly improve the detection performance and decision-making capabilities of the system. The theoretical foundation of text embedding methods lies in distributed representation learning, which captures semantic features by learning the contextual information of words or sentences. A classic text embedding method is Word2Vec, which uses two main models (CBOW and Skip-gram) for word embedding representation. The CBOW model predicts the target word based on the surrounding context, and its objective function is to maximize the following probability:

(14) $$P\left(w_{t}|w_{t-k},\ldots ,w_{t-1},w_{t+1},\ldots ,w_{t+k}\right)=\frac{\exp(v_{w_{t}}^{⊤}\cdot u_{c})}{\sum_{w\in V}\exp\left(v_{w}^{⊤}\cdot u_{c}\right)}$$

where $v_{w_{t}}$ is the embedding vector of the target word $w_{t}$, $u_{c}$ is the average vector of the context window, *V* is the vocabulary, and *k* is the context window size. The Skip-gram model reverses the operation, predicting the context words based on the target word, and its objective function is

(15) $$P\left(w_{t-k},\ldots ,w_{t-1},w_{t+1},\ldots ,w_{t+k}|w_{t}\right)=\prod_{-k\leq j\leq k,j\neq 0}\frac{\exp(v_{w_{t}}^{⊤}\cdot u_{w_{t+j}})}{\sum_{w\in V}\exp\left(v_{w_{t}}^{⊤}\cdot u_{w}\right)}$$

By optimizing these objective functions, Word2Vec generates word vectors that capture semantic relationships, ensuring that semantically similar words are located closer together in the embedding space. In agricultural disease detection, text data embedding not only provides a powerful tool for single-modality text analysis but also lays the foundation for multimodal fusion. The extraction of semantic information from text can provide important contextual knowledge for image data. For example, an image may display spots on a leaf, but visual features alone may not be sufficient to distinguish between types of diseases. However, by combining descriptive text (e.g., ‘The spots on the leaf are round and gradually expand’), the diagnostic accuracy of the model can be significantly improved. The multimodal fusion of text and image data typically requires aligning the features of both modalities. Let the text embedding be $F_{t}\in R^{d}$ and the image features be $F_{v}\in R^{d}$. The fusion representation of both modalities can be achieved by simple weighted fusion:

(16) $$F_{m}=\alpha \cdot F_{v}+\left(1-\alpha \right)\cdot F_{t}$$

where α∈[0,1] is a tunable parameter that controls the weight of each modality. Moreover, attention mechanisms can be employed to further enhance the fusion process. Let the fusion weights be $W\in R^{d\times d}$. The weighted representation of the aligned multimodal features can be expressed as

(17) $$F_{m}=\mathrm{Softmax}\left(F_{v}^{⊤}\cdot W\cdot F_{t}\right)\cdot F_{v}$$

This alignment mechanism allows the fusion process to emphasize the most relevant features from each modality, improving the overall performance of the system. The application of text embedding in multimodal systems not only extends the model’s perceptual ability but also enhances its diagnostic capabilities for complex disease scenarios. For instance, in rare or co-occurring disease cases, the contextual descriptions provided by the text data can effectively supplement the visual information, reducing the model’s misclassification rate. Furthermore, the semantic generation capabilities of text embedding models also offer valuable support for disease prediction and prevention strategies.

### 3.3. Dataset Balance

In this study, addressing the dataset balance issue was crucial for ensuring the model’s performance across various disease categories. Agricultural disease datasets often suffer from class imbalance, where some diseases are more prevalent than others, leading to biased model predictions. To mitigate this, we employed multiple strategies. During the data collection phase, we made efforts to collect a balanced number of samples from different disease types, ensuring diversity and sufficient representation for each class. Additionally, data augmentation techniques like CutMix and GridMask were applied to enhance the minority disease categories, creating synthetic samples and preventing the model from overfitting to more common diseases. Moreover, we adjusted the loss function by incorporating class weights, giving more importance to underrepresented classes during training. These combined efforts helped to maintain a balanced dataset, allowing the model to effectively learn from all classes and improve its generalization ability.

### 3.4. Proposed Method

#### 3.4.1. Overall

**Figure 2:**
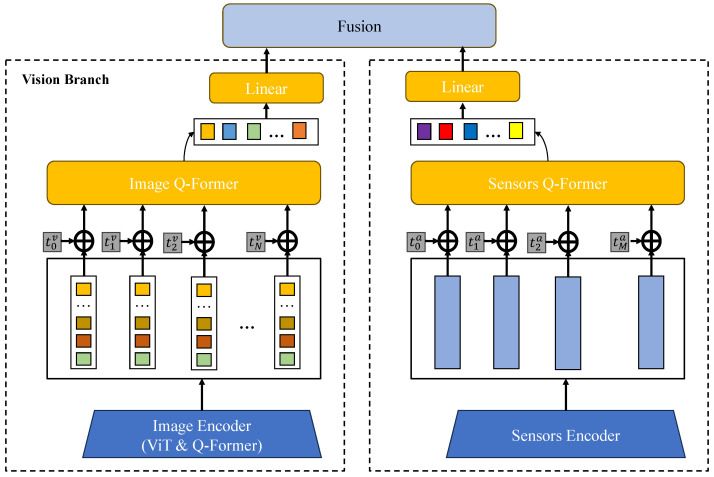
Overall process diagram of the disease detection model. This figure illustrates the eggplant disease detection framework based on multimodal data fusion and embedding attention mechanisms. The Vision Branch processes the image data using the Image Q-Former and Image Encoder (ViT and Q-Former), where the ViT refers to the Vision Transformer, a deep learning architecture used for image processing, and the Q-Former is a specific module designed to transform image features into a suitable format for attention mechanisms. The Sensors Branch processes the sensor data using the Sensors Q-Former and Sensors Encoder, where the Sensors Q-Former transforms the sensor data into a format compatible with attention mechanisms, and the Sensors Encoder is a neural network module that encodes these features for further analysis. The final fusion of both modalities is carried out in the Fusion section, where both image and sensor features are combined for disease detection, allowing the model to effectively use multimodal data.

As shown in Figure 2, the proposed method for eggplant disease detection based on multimodal data and attention mechanisms aims to enhance detection accuracy and robustness by deeply integrating image and sensor data, along with attention mechanisms and embedding loss functions. As shown in previous studies, this method encompasses the entire process from data input to final output, including image feature extraction, sensor data encoding, multimodal fusion, embedding attention mechanisms, and loss function optimization. Initially, preprocessed image and sensor data enter the model’s input module. Image data undergoes processing by CNN, where convolution layers effectively extract fine-grained features of disease areas, such as the color, shape, and texture of lesions. Simultaneously, sensor data are passed through the sensor data encoding module, where the data are embedded using BERT (or another natural language processing model). Following this, the image features and sensor data features enter the multimodal fusion module (Section 3.4.2), which combines these features through weighted concatenation. The fused multimodal features are then processed by the embedding attention mechanism (Section 3.4.3). In this module, attention mechanisms dynamically adjust the model’s focus by calculating the relative importance of different feature parts. After processing by the embedding attention mechanism, the feature vector $F_{m}^{\prime }$ is passed to the loss function module (Section 3.4.4). In this module, a combined loss function, incorporating both image and sensor data features, is optimized to improve the model’s classification accuracy of diseases. Finally, the model, optimized by the loss function, outputs predictions of disease types along with their associated confidence levels. Through multimodal data fusion and the embedding of attention mechanisms, this method effectively addresses challenges such as insufficient single-modal information and high computational complexity inherent in traditional methods, while enhancing the model’s ability to identify different types of diseases, making it highly applicable in practical scenarios.

#### 3.4.2. Multimodal Fusion Embedding Module

**Figure 3:**
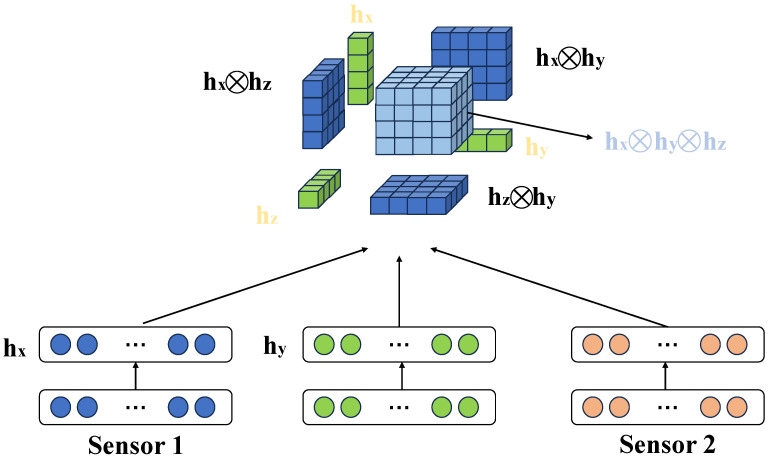
Illustration of multimodal fusion. This figure demonstrates the weighted concatenation process of image features and sensor data features in the multimodal fusion embedding module. Image features are extracted using a CNN (convolutional neural network), a commonly used deep learning model for image processing, while sensor data features are encoded using a model. The weighted concatenation involves the combination of image features ($h_{x}$) and sensor features ($h_{y}$), where each feature is processed and transformed in the fusion module to enhance the model’s ability to detect diseases under different environmental conditions. The concatenation of these features, denoted by $h_{x}⊗h_{z}$, $h_{y}⊗h_{z}$, and other combinations, helps the model to learn from both visual and sensor data simultaneously.

In this study, the proposed multimodal fusion embedding module is one of the core components of the model, designed to efficiently fuse data from different modalities, specifically image and sensor data, thereby enhancing the model’s ability to detect and classify diseases. As shown in Figure 3, since image and sensor data have distinct information representations, effectively combining them in a shared space becomes critical for improving model accuracy. Initially, image data are processed through a CNN for feature extraction. Image data typically contains visual information related to diseases, such as the shape, color, and texture of lesions, which the CNN is capable of capturing at both local and global levels. In this study, the image input is first preprocessed and then fed into a ResNet-50 network for feature extraction. ResNet-50 is a classic deep convolutional neural network consisting of 50 layers, and its design with residual blocks effectively avoids the vanishing gradient problem, improving the extraction of deeper features. After multiple convolutional, pooling, and residual operations, the output image feature $F_{v}\in R^{H_{v}\times W_{v}\times C_{v}}$ is produced, where $H_{v}$ and $W_{v}$ represent the height and width of the image feature map, and $C_{v}$ represents the number of channels, indicating fine-grained features of the image. Meanwhile, sensor data are encoded using a BERT model. Sensor data usually includes disease descriptions and symptom information, which are crucial for accurate disease diagnosis. The BERT model, based on the Transformer architecture, effectively captures contextual relationships between words in the sensor data. After passing through the BERT model, the resulting sensor data feature is $F_{t}\in R^{H_{t}\times W_{t}\times C_{t}}$, where $H_{t}$ and $W_{t}$ represent the height and width of the sensor data feature, and $C_{t}$ is the dimension of the sensor data embedding vector. Through the BERT model, sensor data are transformed into high-dimensional vector representations that incorporate disease background knowledge and expert opinions.

After processing by their respective networks, the image features $F_{v}$ and sensor data features $F_{t}$ enter the multimodal fusion embedding module. In this module, the features are fused through weighted concatenation, a common technique for multimodal data fusion. By assigning different weights to the features from different modalities, this approach balances the contribution of image and sensor data. The advantage of this process is that it allows the model to automatically learn the optimal fusion ratio of image and sensor data features based on the specific task. When the image information is sufficiently rich, the weight α for the image features is larger; when the sensor data provide crucial background knowledge, the weight (1−α) for the sensor data is relatively larger. Through this flexible fusion strategy, the model can automatically adapt to different disease detection scenarios, maximizing the utilization of each modality’s information. After fusion, the resulting multimodal feature $F_{m}$ is passed through a fully connected layer for dimensionality reduction and optimization. The fully connected layer applies a weight matrix $W_{f}$ and bias term $b_{f}$ to perform a linear transformation on the fused feature and adds nonlinear expressiveness through a ReLU activation function. The calculation process is as follows:

(18) $$F_{m}^{\prime }=\mathrm{ReLU}\left(W_{f}\cdot F_{m}+b_{f}\right)$$

where $F_{m}^{\prime }$ is the processed feature after passing through the fully connected layer, which is then used as input to the subsequent classification module for disease classification prediction. The output dimension of the fully connected layer is designed to match the classification task’s requirements, typically reduced to a lower dimension to avoid overfitting and accelerate the training process. The design of the multimodal fusion embedding module has several significant advantages. Firstly, the weighted concatenation fusion method dynamically adjusts the weight according to the importance of each modality’s features, allowing the model to fully leverage the information from each modality in different disease detection scenarios. Image data provide intuitive visual information, while sensor data offer richer background knowledge. The combination of both can significantly improve the ability to identify complex diseases. Secondly, through the feature extraction capabilities of BERT and ResNet-50, both image and sensor data can be processed at a low computational cost, enabling high-quality feature extraction that supports the subsequent classification task. Additionally, through the fully connected layer and ReLU activation function design, the model can effectively map and optimize the feature space, preventing the redundancy of high-dimensional features while improving the training efficiency of the fused features. Through such a design, the proposed method can efficiently handle complex agricultural disease detection tasks, especially when faced with complex scenarios where multiple diseases coexist, exhibiting strong adaptability and accuracy.

#### 3.4.3. Embedding Attention

**Figure 4:**
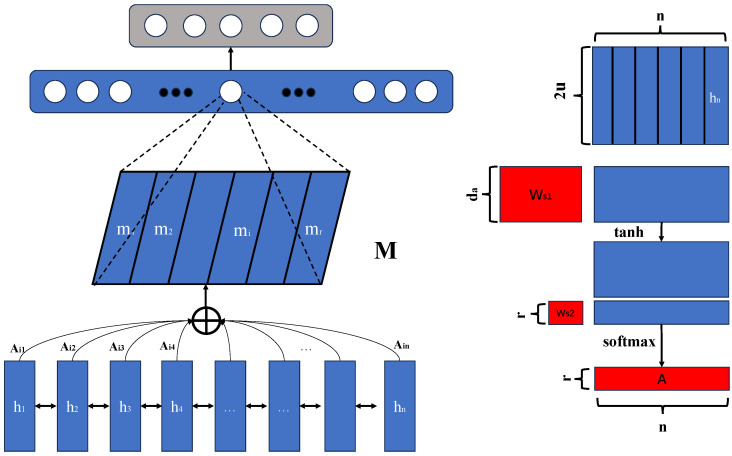
Illustration of the embedding attention mechanism. This figure demonstrates the application of the embedding attention mechanism in multimodal disease detection. The image features and sensor data features are first weighted and concatenated in the multimodal fusion embedding module. Then, the fused features are input into the embedding attention mechanism module, which uses attention to focus on the most relevant features, where $h_{1},h_{2},\ldots ,h_{n}$ represent the concatenated features from the image and sensor data. The output of the attention mechanism, denoted by *A*, helps the model prioritize important features and suppress irrelevant ones, enhancing the detection performance for plant diseases.

The embedding attention mechanism is a widely used technique in multimodal learning, aiming to improve the model’s ability to focus on key information by dynamically allocating importance weights to different features. As shown in Figure 4, unlike traditional self-attention mechanisms, embedding attention not only focuses on the features of the data itself but also addresses how to dynamically adjust the influence between different modalities in the feature space. While self-attention is generally used to capture dependencies within different parts of the same modality, embedding attention extends to multimodal learning by integrating data from modalities such as images and sensor data, enhancing the model’s performance in complex tasks. The primary idea behind embedding attention is to assign a weight to each input feature based on the correlation between the features, where this weight reflects the contribution of the feature to the final task. In design, embedding attention combines feature representations from image and sensor data modalities and uses self-attention to dynamically adjust the weights between these modalities. Specifically, when image and sensor data features are fused through the multimodal fusion embedding module, embedding attention calculates the importance of each feature based on these fused features, thereby optimizing the feature representation. This allows the model not only to enhance its learning capability for important regions but also to minimize interference from irrelevant information during training, improving the model’s sensitivity to key disease features.

The implementation of embedding attention is based on the core principle of self-attention but further improves the model’s complexity and expressive power by introducing multimodal information fusion. The network architecture includes several self-attention layers, where each layer computes the relationship between image and sensor data features, generating attention weights and applying them to the feature vectors. During the operation of each layer, the input feature vector is linearly transformed to generate query, key, and value matrices (Q, K, V). These matrices come from the feature representations of image and sensor data, and after applying the attention weighting, they are fused with the original features. Ultimately, through the stacking of multiple attention layers, the model gradually adjusts the representation of each feature, making it more aligned with the task requirements. The mathematical formulation of this mechanism can be described as follows. Let the input feature be the fused feature $F_{m}$ from the image and sensor data modalities, where $F_{m}\in R^{H\times W\times C}$, with *H* and *W* being the height and width of the feature map and *C* being the number of channels. Through linear transformation, query, key, and value matrices are generated:

(19) $$Q=W_{q}F_{m},\;K=W_{k}F_{m},\;V=W_{v}F_{m}$$

Here, $W_{q}$, $W_{k}$, and $W_{v}$ are learnable weight matrices, and Q, K, and V are the query, key, and value matrices, respectively. Then, the similarity between the query and key matrices is computed, and the attention weights are obtained using the softmax function. By calculating the weight for each feature, the model can allocate appropriate attention to the fused image and sensor data features. Ultimately, the weighted feature vector $F_{m}^{\prime }$ is passed to the subsequent module for further processing and prediction. Unlike the traditional self-attention mechanism, embedding attention introduces the fusion of multimodal data features, enabling the model to not only focus on the local importance of image features but also to effectively capture the contextual relationships in the sensor data features. This design allows the model to adaptively adjust the weight between image and sensor data, thus improving its ability to identify disease types and lesion areas. By jointly weighting multimodal features, embedding attention enhances both the detection accuracy and robustness of the model, especially in cases where multiple diseases coexist or when data are missing, significantly improving detection performance.

#### 3.4.4. Embedding Loss

In this study, a novel embedding loss function is proposed to optimize the joint feature representation learning of multimodal data (image and sensor data). The design of embedding loss differs from traditional loss functions, such as cross-entropy loss or MSE loss. Traditional loss functions typically focus on the classification or regression tasks of a single modality of data (such as image or sensor data) and generally do not consider the joint learning of multimodal information or the relationships between multimodal data. The embedding loss, however, aims to optimize the feature representations of both image and sensor data modalities, allowing these modalities to be effectively fused in a shared space, thereby improving the model’s performance in disease detection tasks. In traditional loss functions, such as cross-entropy loss, the difference between predicted and actual categories is directly computed. The formula is

(20) $$L_{\mathrm{CE}}=-\frac{1}{N}\sum_{i=1}^{N}\sum_{c=1}^{C}y_{i,c}\log{\hat{y}}_{i,c}$$

where $y_{i,c}$ is the true label of the *i*-th sample, and ${\hat{y}}_{i,c}$ is the predicted probability of the model. Cross-entropy loss updates the model’s parameters by measuring the discrepancy between the prediction and the actual label, commonly used for classification problems. However, cross-entropy loss performs suboptimally in multimodal learning because it does not consider the information fusion between image and sensor data modalities, nor does it address the relationships between these modalities in the joint feature space. To address this issue, this study proposes embedding loss, which aims to optimize the joint embedding representation of image and sensor data modalities, allowing information from different modalities to be effectively aligned in a shared feature space. In the framework of embedding loss, image and sensor data features are jointly learned through the multimodal fusion embedding module. Let the image features be $F_{v}\in R^{H_{v}\times W_{v}\times C_{v}}$ and the sensor data features be $F_{t}\in R^{H_{t}\times W_{t}\times C_{t}}$, and after weighted concatenation, the joint feature $F_{m}$ is obtained. The weighted concatenation of image and sensor data features results in the joint feature $F_{m}$, which is then used as input for further network processing. The core idea of embedding loss is to effectively align image and sensor data in the joint feature space, ensuring that features of the same category are as close as possible, while features of different categories are as far apart as possible. Therefore, the embedding loss calculation relies not only on the correctness of classification but also on the alignment of features between modalities. Specifically, embedding loss consists of two parts: the alignment loss between image and sensor data features and the classification loss. The alignment loss between image and sensor data features can be measured by calculating the distance between their embeddings. Let the embeddings of image and sensor data be $F_{v}$ and $F_{t}$, respectively, and the alignment loss can be measured using cosine similarity, expressed as

(21) $$L_{\mathrm{align}}=1-\frac{F_{v}\cdot F_{t}}{|F_{v}\left|\right|F_{t}|}$$

This loss function measures the cosine similarity between the image and sensor data features in the embedding space. A higher similarity indicates better alignment of image and sensor data features in the joint representation space, while a lower similarity indicates poorer alignment. In addition to the alignment loss, embedding loss also includes a traditional classification loss, such as cross-entropy loss, to ensure that the final classification result is as close as possible to the true label. Ultimately, the embedding loss can be expressed as the weighted sum of the image and sensor data alignment loss and the classification loss, as shown below:

(22) $$L_{\mathrm{embedding}}=L_{\mathrm{CE}}+\lambda \cdot L_{\mathrm{align}}$$

where λ is the weighting coefficient used to balance the classification loss and alignment loss. By optimizing the embedding loss, the model can improve classification accuracy while optimizing the alignment of image and sensor data features in the joint feature space, thereby enhancing the overall performance of disease detection. Compared with traditional single-loss functions, embedding loss has significant advantages. First, embedding loss fully utilizes the complementary information between image and sensor data, optimizing the joint representation of features from both modalities and improving the model’s multimodal learning capability. Second, by simultaneously optimizing the alignment degree and classification accuracy of image and sensor data features, embedding loss enables better handling of complex disease detection tasks, particularly in cases of multiple diseases coexisting or missing data, making the model more robust in inference. Through this approach, embedding loss effectively addresses the information asymmetry and alignment issues in multimodal learning, providing stronger model performance and broader application prospects for eggplant disease detection tasks.

### 3.5. Experimental Setup

#### 3.5.1. Hardware and Software Platform

In this study, a high-performance computing cluster was chosen as the experimental hardware platform to ensure efficient processing of multimodal data and complex deep learning models. The main hardware includes servers equipped with NVIDIA A100 GPUs, each with 40 GB of memory, capable of supporting large-scale model training and inference tasks. Additionally, computing nodes with AMD EPYC processors, featuring 128 cores, were utilized for data preprocessing and model optimization tasks that require high computational load. High-speed NVMe SSD storage was employed to provide fast read and write support for data loading and model checkpoint saving. The network interconnection adopted InfiniBand technology, enabling low-latency, high-bandwidth data transmission between multiple nodes to meet the demands of distributed training.

The software platform was configured with a deep learning development environment based on the Ubuntu 22.04 operating system, integrating two mainstream deep learning frameworks, PyTorch 2.0 and TensorFlow 2.13, to ensure model development flexibility and compatibility. Dependency management was achieved through the conda environment, ensuring library version consistency and experimental reproducibility. For data processing, tools such as OpenCV 4.1.0.25 and Pandas 1.1.0 were used to facilitate image augmentation and multimodal data feature alignment analysis. During model training, GPU acceleration was supported by CUDA 12.0 and cuDNN 8.9, significantly improving computational efficiency. Furthermore, the Weights & Biases (W&B) platform was employed for experimental result visualization and tracking management, facilitating model performance comparison and parameter tuning. The combination of these hardware and software configurations provided robust technical support for the efficient progress of this research.

#### 3.5.2. Optimizers and Hyperparameters

In this study, the dataset was split into training, validation, and test sets to ensure a comprehensive evaluation of the model’s performance during training, validation, and testing. Specifically, the dataset was divided into 70% for training, 15% for validation, and 15% for testing, with the training set used for model parameter learning, the validation set for hyperparameter tuning and model selection, and the test set for evaluating model generalization on unseen data. To further reduce the impact of data partitioning on the results, 5-fold cross-validation was applied, where the dataset was evenly split into five subsets, with one subset chosen as the validation set and the remaining four subsets used for training. This process was repeated five times, and the final model performance was obtained by averaging the results from all folds.

For model optimization, the Adam optimizer was employed with an initial learning rate of η=0.001, and a cosine annealing learning rate scheduling strategy was applied to dynamically adjust the learning rate. A weight decay coefficient of $\lambda =1\times 10^{-4}$ was used to mitigate overfitting and accelerate model convergence. During training, the batch size was set to 32, and data were randomly shuffled in each iteration to improve the robustness of training.

### 3.6. Evaluation Metrics

In agricultural disease detection tasks, multiple evaluation metrics are typically used to assess the model’s performance comprehensively, including precision (*P*), recall (*R*), accuracy (Acc), and mean average precision (mAP). In this study, particular attention was paid to mAP@75, the mean average precision at an IoU threshold of 0.75, to assess the model’s detection capability under a high standard. *P* measures the accuracy of the model’s predictions, i.e., how many of the predicted positive samples are actually positive. Its formula is:

(23) $$P=\frac{TP}{TP+FP}$$

where TP denotes the number of true positive samples (correctly predicted as positive) and FP denotes the number of false positive samples (incorrectly predicted as positive). A high precision indicates higher confidence in the model’s positive predictions. *R* reflects the model’s completeness, i.e., how many of the actual positive samples are correctly predicted as positive. Its formula is:

(24) $$R=\frac{TP}{TP+FN}$$

where FN denotes the number of false negative samples (incorrectly predicted as negative). A high recall indicates the model’s ability to capture more positive samples, though it may lead to more false positives. Acc is an overall performance metric that measures the proportion of correctly predicted samples, i.e., the ratio of correct predictions to total samples. Its formula is

(25) $$Acc=\frac{TP+TN}{TP+TN+FP+FN}$$

where TN represents the number of true negative samples (correctly predicted as negative). Accuracy provides an intuitive measure of the model’s overall performance but may lose its reference value when the sample class distribution is imbalanced. mAP is a commonly used metric in object detection tasks to evaluate the model’s performance at different confidence thresholds. It is calculated by computing AP for each class and averaging over all classes. For a single class, AP is computed based on the precision–recall curve, as defined by the following formula:

(26) $$AP=\int_{0}^{1}P\left(R\right)dR$$

where P(R) represents the relationship between precision and recall. The mAP is then the average of all class-specific AP values:

(27) $$mAP=\frac{1}{C}\sum_{c=1}^{C}AP_{c}$$

where *C* is the total number of classes, and $AP_{c}$ is the average precision for the *c*-th class. In this study, particular attention was paid to mAP@75, which measures the average precision at an IoU threshold of 0.75. These evaluation metrics play an important role in agricultural disease detection tasks. Precision and recall help balance prediction accuracy and completeness, accuracy provides an intuitive measure of overall performance, and mAP and mAP@75 assess the model’s comprehensive capability in object detection tasks.

### 3.7. Baseline Models

To evaluate the effectiveness and competitiveness of the proposed method, several classic object detection models were chosen as baseline methods, including RetinaNet [46], SSD [47], LeafDetection [48], YOLOv8 [49], and DETR [50]. These baseline models have distinct characteristics and have demonstrated excellent detection performance in various scenarios. RetinaNet introduces the focal loss function to address class imbalance issues, effectively reducing the impact of negative samples on the loss. SSD (Single Shot MultiBox Detector) achieves a balance between real-time performance and accuracy through multiscale feature detection, using a multilayer feature pyramid to capture objects of different sizes. The LeafDetection model is specifically designed for agricultural scenarios, combining domain knowledge and lightweight network structures to excel in leaf detection tasks. YOLOv8, the latest version in the YOLO series, significantly improves detection speed and accuracy with dynamic receptive fields and adaptive anchor box mechanisms. DETR (DEtection TRansformer) introduced the Transformer architecture for the first time in object detection, using self-attention mechanisms to directly predict the global relationships of target boxes. Its loss function consists of classification loss and bounding box loss. These baseline models provide a comprehensive and rigorous performance comparison, and their results allow for further validation of the advantages and improvement directions of the proposed method.

## 4. Results and Discussion

### 4.1. Disease Detection Results

**Figure 5:**
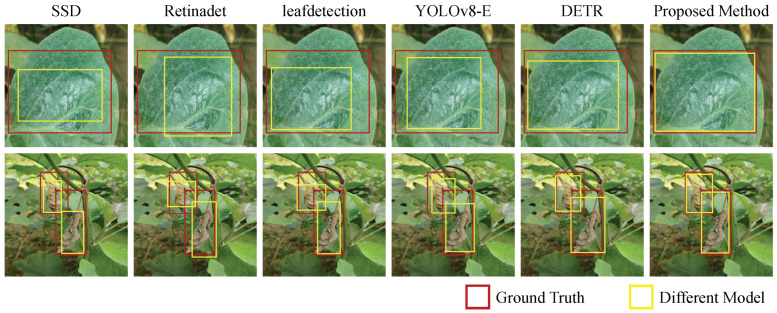
Visualization results of different models.

The aim of this experiment is to evaluate the performance of different disease detection models in the eggplant disease detection task. As shown in Table 2 and Table 4, by comparing the models based on performance metrics such as precision, recall, accuracy, and mAP@75 (mean average precision at IoU threshold of 0.75), the strengths and weaknesses of each model can be thoroughly analyzed, enabling a better understanding of their applicability in disease detection and revealing the theoretical advantages of different models. According to the experimental results, YOLOv8 performs the best among all models, particularly excelling in mAP@75 with a high score of 0.98, which indicates excellent performance in bounding box localization and precision. YOLOv8 is a real-time object detection model based on deep learning, utilizing dynamic receptive fields and adaptive anchor box mechanisms, allowing it to rapidly focus on disease regions in more complex backgrounds while optimizing the framework to enhance the detection accuracy of small target diseases. The Retinadet and leafdetection models excel in recall (0.83, 0.84) and accuracy (0.85, 0.86), respectively, making them suitable for tasks that require fine-grained feature extraction. The leafdetection model, specifically designed for agricultural scenarios, integrates domain prior knowledge and lightweight network structures, achieving good results even in environments with limited computational resources. DETR adopts a Transformer architecture and uses global self-attention mechanisms to process object detection problems. Although its accuracy and recall (0.92 and 0.90, respectively) are relatively high, its performance in mAP@75 (0.91) is slightly lower than YOLOv8, indicating slightly lower precision in bounding box localization. In contrast, the proposed method achieves 0.94 in precision and 0.90 in recall, demonstrating comparable high accuracy to DETR while maintaining a good mAP@75 of 0.91, indicating that the proposed method strikes a balance between classification accuracy and target localization accuracy, offering comprehensive advantages in both classification and localization performance (Figure 5).

From a mathematical perspective, the advantage of YOLOv8 comes from its efficient target localization mechanism and end-to-end training, enabling it to optimize precision and recall to the highest extent when processing images rapidly. Retinadet and leafdetection, on the other hand, achieve good adaptation to small sample sizes through focal loss and lightweight network structures, making them suitable for applications in low-computation environments. DETR, by incorporating a self-attention mechanism, is capable of capturing more complex feature relationships, particularly suited for scenarios that require global information processing. However, its performance in bounding box precision is slightly inferior to that of YOLOv8. The proposed method, which integrates multimodal data and attention mechanisms, leverages the advantages of information to improve detection precision and robustness while addressing challenges in multimodal data processing. Through optimized feature representation and attention mechanisms, the proposed method significantly enhances the overall performance of the model, achieving balanced optimization across all metrics.

### 4.2. Results for Different Diseases Using the Proposed Method

The purpose of this experiment is to evaluate the performance of the proposed disease detection method on different types of diseases, verifying the method’s adaptability and robustness in handling multiple diseases. As shown in Table 3, the experiment compares the performance of the model on various diseases based on multiple metrics, providing a comprehensive analysis of its performance. This experiment not only demonstrates the model’s advantages in multidisease detection but also reveals its specific applicability and effectiveness in recognizing different diseases. To address the concern raised about testing the model on other diseases, we extended our experiment to include additional diseases. Although the YOLO model performs similarly to our proposed model for certain diseases, we sought to evaluate its performance across a broader range of diseases, including those where our approach might have an edge. By expanding the experiment to include additional diseases, we could assess the strengths of our approach in comparison with YOLO across different disease types, especially in cases where multimodal fusion and attention mechanisms may enhance performance. From the experimental results in the table, bacterial leaf spot disease achieved the best performance with a precision of 0.97, recall of 0.92, accuracy of 0.94, and mAP@75 of 0.93. The high precision and recall for bacterial leaf spot disease indicate that the model is able to accurately identify most real cases and almost entirely avoids false negatives. This may be attributed to the characteristic features of bacterial leaf spot disease, such as the distinct color and shape of the lesions, allowing the model to effectively differentiate this disease based on visual features.

Among all the diseases, leaf mold and fusarium wilt also performed relatively well, with precision values of 0.96 and 0.94 and recall values of 0.91 and 0.90, respectively. This suggests that the model achieves high accuracy and coverage for these diseases as well. In contrast, brown spot and brown stripe diseases performed relatively lower, but their precision and recall values were still above 0.91 and 0.88, respectively, showing good detection capability. The performance for these diseases, particularly brown spot and brown stripe, highlights the challenges in detecting diseases with more varied symptoms or subtle differences. Our method, which combines image and sensor data with attention mechanisms, showed superior detection capability in these complex cases. Overall, all diseases had high mAP@75 values, indicating that the proposed method also performs robustly in target localization. In comparison with YOLO, our method exhibits a notable advantage in more complex cases, where multimodal data fusion and attention-based feature weighting improve detection accuracy.

From a theoretical perspective, the model’s performance on different diseases is closely related to the disease’s visual features, the model’s processing capability, and the diversity of the dataset. Bacterial leaf spot disease, with its distinct lesion characteristics, allows the model to easily recognize the disease based on visual features. In contrast, brown spot and brown stripe diseases, due to the diversity in lesion shape and similarity in color, require stronger feature discrimination for accurate recognition. The deep learning model used in this study, by combining multimodal information and attention mechanisms, enables the model to focus on key disease features, particularly in complex disease types, allowing it to effectively extract complementary information from both modalities to enhance detection capabilities. The differences in disease features likely cause slight performance variations in some diseases, which are closely related to feature selection and weighting mechanisms during the model’s training and learning process. Mathematically, the embedding attention mechanism of the proposed method dynamically adjusts the weight between modalities in the multimodal feature space, which plays a critical role in improving recognition ability for complex diseases. For diseases such as bacterial leaf spot, which are easy to distinguish, the model makes full use of significant visual features, while for diseases like brown spot and brown stripe, the information provides background knowledge and descriptions that help the model make accurate classifications and detections. By optimizing the embedding loss, the model effectively learns in the joint representation space of multimodal data, thereby improving overall detection accuracy and robustness.

### 4.3. Ablation Study of Different Attention Mechanisms

The purpose of this experiment was to evaluate the impact of different attention mechanisms on the performance of disease detection models through an ablation study. As shown in Table 4, by comparing the performance of the standard self-attention, Convolutional Block Attention Module (CBAM), and embedding attention mechanisms across various metrics, the goal was to gain a deeper understanding of the applicability and advantages of these attention mechanisms in disease detection tasks. The core objective of this experiment was to verify whether the embedding attention mechanism could provide a greater improvement in model performance compared with other commonly used attention mechanisms. According to the experimental results, the standard self-attention mechanism performed relatively poorly, with precision at 0.73, recall at 0.70, accuracy at 0.72, and mAP@75 at 0.71. In contrast, CBAM showed a performance improvement, with precision reaching 0.85, recall at 0.81, accuracy at 0.83, and mAP@75 at 0.82. The embedding attention mechanism outperformed all models, with precision at 0.94, recall at 0.90, accuracy at 0.92, and mAP@75 at 0.91.

**Table 4:** Ablation Study of Different Attention Mechanisms.

Model	Precision	Recall	Accuracy	mAP@75
Standard Self-Attention	0.73	0.70	0.72	0.71
CBAM	0.85	0.81	0.83	0.82
Embedding Attention	0.94	0.90	0.92	0.91

This result demonstrates that while the self-attention mechanism enhances the model’s expressive power by capturing global dependencies between input features, the standard self-attention mechanism, when used in isolation, might generalize attention too broadly, failing to effectively focus on key information related to the disease detection task. Although CBAM effectively addresses the attention distribution for both channel and spatial information, its performance in disease detection still leaves room for significant improvement compared with the embedding attention mechanism. The embedding attention mechanism combines the global dependency modeling capability of self-attention with the integration of multimodal data, enabling the model to dynamically adjust and align the weights between different modalities. This design allows the model to better focus on the critical features in the disease regions while avoiding overemphasis on irrelevant information. Especially in the case of complex or varying disease images, the embedding attention mechanism excels by weighting different features, achieving impressive performance in both accuracy and recall.

### 4.4. Ablation Study of Different Loss Functions

The objective of this experiment was to evaluate the effect of different loss functions on the performance of the disease detection model, particularly in handling multimodal data. As shown in Table 5, by comparing the performance of cross-entropy loss, focal loss, and embedding loss across various metrics, the goal was to analyze the strengths and weaknesses of each loss function and assess their contribution and role in model training. This experiment aimed to validate whether the embedding loss could effectively improve model performance, especially in the context of multimodal data processing. The results of the experiment show that when using cross-entropy loss, the model performed relatively poorly, with precision at 0.71, recall at 0.68, accuracy at 0.70, and mAP@75 at 0.69. Focal loss performed better than cross-entropy loss, achieving precision at 0.82, recall at 0.79, accuracy at 0.81, and mAP@75 at 0.80. Embedding loss outperformed all other loss functions, with precision at 0.94, recall at 0.90, accuracy at 0.92, and mAP@75 at 0.91.

The experimental results show that traditional cross-entropy loss has certain limitations when handling multimodal data, especially in the fusion of multiple sources of information. It fails to adequately consider the relationships between modalities, thus affecting the model’s ability to accurately recognize disease features. Cross-entropy loss is more suitable for single-modal classification tasks, but it does not effectively guide the model to learn the collaborative features between modalities for multimodal tasks, leading to suboptimal performance in terms of both precision and recall. Focal loss, by adjusting the weights for easy-to-classify and hard-to-classify samples, performs better in addressing class imbalance. It encourages the model to focus more on difficult samples, preventing overfitting to easy-to-classify samples, which results in improvements in recall and accuracy. However, despite its ability to improve classification performance, its performance in multimodal data fusion still lags behind embedding loss. This indicates that embedding loss can effectively align and weight the multimodal features, enabling the model to better understand the relationships between different modalities, thus enhancing overall detection accuracy. Embedding loss optimizes the joint representation of multimodal data, ensuring that information can be effectively fused in a shared space, thereby improving the performance in disease detection tasks.

From a mathematical perspective, the cross-entropy loss function updates the model parameters by calculating the discrepancy between the predicted and true labels. Its core formula is

(28) $$L_{\mathrm{CE}}=-\frac{1}{N}\sum_{i=1}^{N}\sum_{c=1}^{C}y_{i,c}\log{\hat{y}}_{i,c}$$

where $y_{i,c}$ is the true label for the *i*-th sample, and ${\hat{y}}_{i,c}$ is the predicted probability. This loss function is not ideal for multimodal data because it does not consider the correlation and synergy between features. Focal loss, by introducing a scaling factor γ, reduces the impact of easy-to-classify samples, especially when dealing with hard-to-classify diseases. The formula for focal loss is

(29) $$L_{\mathrm{FL}}=-\alpha {\left(1-{\hat{y}}_{i,c}\right)}^{\gamma }\log{\hat{y}}_{i,c}$$

where α is a balancing factor, and γ is a parameter for adjusting the difficulty of samples. Focal loss increases the weight for difficult samples, effectively enhancing the model’s recall rate for challenging diseases. In comparison, embedding loss not only considers the discrepancy between predicted and true labels but also introduces an alignment loss between features, ensuring that information from both modalities is fused effectively in the shared space. Specifically, the alignment loss is calculated by measuring the similarity between features, as shown in the following formula:

(30) $$L_{\mathrm{align}}=1-\frac{F_{v}\cdot F_{t}}{|F_{v}\left|\right|F_{t}|}$$

where $F_{v}$ and $F_{t}$ are the features, and $|F_{v}|$ and $|F_{t}|$ are their respective magnitudes. By optimizing this alignment loss, embedding loss enables better alignment between features in the joint representation space, thus enhancing the model’s overall performance. Ultimately, embedding loss outperforms traditional cross-entropy loss and focal loss by effectively leveraging multimodal data and ensuring robust feature alignment, improving both detection accuracy and robustness in disease detection tasks.

### 4.5. Impact of Sensor Data on Model Performance

In this study, we conducted an ablation experiment to evaluate the effect of sensor data on the performance of the disease detection model. The experiment was designed to assess three different configurations: using only image data, using only sensor data, and using both image and sensor data combined. This experiment allows us to analyze how each type of data contributes to the model’s performance in terms of key evaluation metrics such as precision, recall, accuracy, mAP@75, and FPS.

As shown in Table 6, this study clearly demonstrates that using both image and sensor data combined provides the best performance across all evaluation metrics. The model’s precision, recall, and accuracy significantly improve when sensor data are incorporated along with image data. While image-only models provide reasonable results, the combination of image and sensor data enhances the model’s ability to detect diseases more accurately and robustly, particularly in complex or variable environmental conditions. This highlights the importance of multimodal data fusion in improving the overall performance of disease detection models.

### 4.6. Impact of Augmentation Methods on Model Performance

In this study, we conducted an ablation experiment to evaluate the impact of different augmentation methods on the performance of the disease detection model. The experiment aimed to assess four different configurations, using CutMix, Mosaic, GridMask, and a combination of CutMix + Mosaic + GridMask. These augmentation techniques are commonly employed to enhance the robustness of models by creating diverse variations in the training data. The results are summarized in the table below, which compares the performance of the model with different augmentation strategies based on key evaluation metrics such as precision, recall, accuracy, and mAP@75.

As shown in Table 7, it is evident that each augmentation method improves the model’s performance, but the combination of CutMix, Mosaic, and GridMask yields the best results. While each method individually improves precision and recall, their combination significantly boosts model performance across all metrics. This confirms that combining multiple augmentation strategies can further enhance the robustness and generalization ability of the disease detection model, making it more effective in real-world applications.

### 4.7. Testing on Other Datasets

The aim of the experimental design was to evaluate the performance of different models on the extended dataset. By comparing the performance of various models in terms of each metric, the advantages and disadvantages of different models in practical applications could be analyzed in depth. From the experimental results in Table 8, the proposed method performed excellently in metrics such as precision, recall, and accuracy, reaching 0.91, 0.87, and 0.89, respectively, demonstrating strong disease recognition ability and good robustness. Meanwhile, its mAP@75 was 0.88, indicating that the method also had a relatively excellent performance in target localization. In addition, the FPS value was 46, suggesting that the method was optimized in terms of inference speed and could operate efficiently in practical applications. From the analysis of the mathematical characteristics of the model, although the SSD model performed well in precision and recall, its complexity was relatively low, and it was unable to fully capture complex disease features. Therefore, its performance in accuracy and mAP@75 was limited. Retinadet performed well in processing local features of images but failed to exert great advantages on more complex disease images. In contrast, the YOLOv8-E and DETR models could better cope with complex disease detection tasks through more powerful network architectures and global information processing. The model proposed in this paper integrated multimodal data and embedded an attention mechanism. Through feature fusion and weighted optimization, it performed outstandingly in multiple evaluation metrics, demonstrating its advantages in the disease detection task.

### 4.8. Deployment of the Disease Control System

In this study, the proposed eggplant disease detection method based on multimodal data fusion and embedding attention mechanism has been successfully deployed on mobile devices, specifically using the Huawei Mate 60 as the experimental platform. To ensure that the model can run efficiently on resource-constrained devices, several lightweight techniques were adopted during the development process, aimed at reducing computational complexity, lowering memory usage, and optimizing inference speed. The model development process, from the training phase to deployment, was carefully planned, including the application of techniques such as model compression, quantization, and hardware acceleration.

The development process began with data preprocessing and model training. During data preprocessing, images and sensor data were converted into formats suitable for model input, and data augmentation techniques were used to expand the sample space, enhancing the model’s generalization capability. Subsequently, deep learning frameworks such as TensorFlow Lite and PyTorch Mobile were used for model training, and standard evaluation metrics (e.g., accuracy, recall, and mAP@75) were employed to assess model performance. During training, the optimization strategies of the embedding attention mechanism and multimodal data fusion modules effectively improved the model’s ability to focus on disease-related features and its capacity to recognize complex diseases.

To adapt the model to run on mobile devices, several lightweight techniques were applied. First, model compression techniques were used to reduce the model’s size and storage requirements. After training the model, pruning was applied to remove those neuron connections with little impact on the prediction results, thus reducing the model’s complexity and the number of parameters. Specifically, for each layer’s weight matrix W, the importance of each connection (e.g., based on the absolute value of the weights) was computed, and those contributing less to the output were removed. This process can be expressed as

(31) $$W^{\prime }=W\cdot I_{|W|>\epsilon }$$

Here, I represents an indicator function, where connections are retained if |W| exceeds the threshold ϵ; otherwise, they are pruned. This approach effectively reduces the number of parameters in the model while maintaining high detection accuracy.

Additionally, quantization techniques were widely applied to accelerate the model’s inference process. By converting the floating-point weights and activation values into fixed-point numbers, quantization significantly reduces the model’s memory footprint and computational overhead. Specifically, if the original weight is W, a floating-point number, after quantization, the weight will be mapped to a smaller integer range:

(32) $$W^{\prime }=\mathrm{round}\left(\frac{W}{\Delta }\right)$$

where Δ is the quantization step size, and $W^{\prime }$ is the quantized integer weight. The quantized model not only reduces memory usage but also benefits from the hardware accelerator (e.g., the Huawei Mate 60’s NPU) for more efficient inference. The quantization step size Δ can be adjusted based on values learned during training, thereby improving inference speed while maintaining precision.

To further enhance inference speed, hardware acceleration techniques were integrated. When deploying the model on the Huawei Mate 60’s NPU, specialized hardware optimizations were used for CNN and attention mechanisms. Through the NPU’s parallel computing capabilities, the model’s inference speed was significantly improved. The key to hardware acceleration lies in the ability to speed up operations in the convolution and attention layers through matrix multiplication, allowing the model to perform large-scale computational tasks in a short amount of time. Specifically, the NPU uses parallel computation and pipelining techniques to decompose large matrix multiplications into smaller subtasks that can be executed concurrently, significantly boosting computational efficiency. In this process, vectorization and hardware-optimized matrix multiplications enabled the model to complete disease detection on images in less than one second.

In conclusion, through the application of model compression, quantization, and hardware acceleration, an efficient eggplant disease detection system was deployed on the mobile platform. This approach enables the method to provide efficient disease recognition even in resource-constrained environments, making it highly applicable in practical agricultural production scenarios.

### 4.9. Limitation and Future Work

Despite the significant results achieved in the experiments of the eggplant disease detection method based on multimodal data fusion and embedded attention mechanisms, several limitations remain. Firstly, although the model performs excellently in terms of accuracy, recall, and mAP@75, the training process is still limited by the size and quality of the dataset. While various disease types and sensor data have been collected for the study, providing a representative and diverse dataset, there is still a scarcity of data for certain specific disease types. Secondly, although the embedded attention mechanism effectively enhances the model’s ability to focus on key information, its computational complexity and training time overhead are relatively high. When handling larger-scale datasets, the model’s complexity increases, and despite the reduction in computational overhead through feature dimensionality reduction and optimization strategies, further research is needed to optimize the computational efficiency of the model while maintaining accuracy. This is crucial for its application in large-scale production environments. Future research can focus on several areas for further optimization. Firstly, data augmentation and transfer learning methods can be employed to expand the dataset, especially for disease types with insufficient data, by adding image and sensor data samples from different environmental conditions, which would further improve the model’s robustness and generalization ability. Secondly, techniques such as deep compression and model distillation can be combined to optimize the embedded attention mechanism and multimodal data fusion modules, reducing the model’s computational complexity and training time and making it more suitable for resource-constrained application scenarios. Finally, with the continuous development of agricultural Internet of Things (IoT) technologies, the types and accuracy of sensors are constantly improving. Future research could leverage additional sensor data, such as soil moisture and light intensity, to further enhance the model’s diagnostic capabilities and improve its practical value in real-world agricultural production.

In future work, we aim to further enhance the applicability of our proposed disease detection model in real-world agricultural scenarios. While the model has demonstrated strong performance in controlled experimental environments, real-world conditions present additional challenges, such as varying environmental factors, different growth stages, and diverse crop management practices. We plan to collect more diverse field data that captures these complexities and evaluate the model’s performance in dynamic, real-world settings. Furthermore, we will explore strategies for real-time deployment, ensuring that the model can function on edge devices with limited computational resources. Additionally, we aim to improve the model’s adaptability to multidisease environments, where multiple diseases might occur simultaneously, and investigate how to integrate real-time feedback for continuous model refinement. These improvements will make the model more robust and practical for large-scale agricultural applications.

## 5. Conclusions

The purpose of this experiment design is to evaluate the impact of different attention mechanisms on the performance of the disease detection model through an ablation study. The core goal of this experiment is to verify whether the embedding attention mechanism can bring greater improvements in model performance compared with other commonly used attention mechanisms. According to the experimental results, the embedding attention mechanism outperformed all other models, achieving a precision of 0.94, recall of 0.90, accuracy of 0.92, and mAP@75 of 0.91. This result indicates that while self-attention can enhance the model’s representational ability by capturing global dependencies among input features, using standard self-attention alone may lead to overly generalized feature attention, preventing effective focus on key information related to disease detection tasks. Although CBAM handles the attention distribution of channel and spatial information better, its performance in disease detection still has substantial room for improvement compared with the embedding attention mechanism. The embedding attention mechanism combines the global dependency modeling ability of self-attention while incorporating multimodal data to enable dynamic weighting and feature alignment between different modalities. This design allows the model to focus better on the key features of the disease regions while avoiding excessive attention to irrelevant information. Particularly in the case of complex or variable disease images, the embedding attention mechanism can adjust the weighting of different features, leading to excellent performance in both accuracy and recall.

## Figures and Tables

**Table 1:** Number of images for different diseases.

Disease	Data
Brown spot	792
Brown stripe	1703
Fusarium wilt	1194
Leaf mold	1386
Bacterial leaf spot	1583

**Table 2:** Experimental results of disease detection models.

Model	Precision	Recall	Accuracy	mAP@75	FPS
SSD	0.83	0.80	0.82	0.82	36
Retinadet	0.85	0.83	0.85	0.85	29
leafdetection [48]	0.87	0.84	0.86	0.85	35
YOLOv8-E [2]	0.90	0.87	0.89	0.89	42
DETR	0.92	0.90	0.91	0.91	25
**Proposed Method**	**0.94**	**0.90**	**0.92**	**0.91**	**50**

**Table 3:** Experimental results of different diseases using the proposed method and comparison with YOLO.

Disease	Precision	Recall	Accuracy	mAP@75
Brown spot disease (YOLO)	0.88	0.85	0.86	0.85
Brown stripe disease (YOLO)	0.89	0.87	0.88	0.87
Fusarium wilt (YOLO)	0.91	0.88	0.89	0.89
Leaf mold (YOLO)	0.92	0.90	0.91	0.90
Bacterial leaf spot (YOLO)	0.94	0.91	0.93	0.91
Brown spot disease (Proposed)	0.91	0.88	0.89	0.88
Brown stripe disease (Proposed)	0.93	0.89	0.90	0.89
Fusarium wilt (Proposed)	0.94	0.90	0.91	0.91
Leaf mold (Proposed)	0.96	0.91	0.93	0.92
Bacterial leaf spot (Proposed)	0.97	0.92	0.94	0.93

**Table 5:** Ablation Study of Different Loss Functions.

Model	Precision	Recall	Accuracy	mAP@75
Cross-Entropy Loss	0.71	0.68	0.70	0.69
Focal Loss	0.82	0.79	0.81	0.80
Embedding Loss	0.94	0.90	0.92	0.91

**Table 6:** Impact of image and sensor data on disease detection model.

Model	Precision	Recall	Accuracy	mAP@75	FPS
Image	**0.85**	**0.82**	**0.84**	**0.83**	**51**
Sensor	**0.79**	**0.82**	**0.80**	**0.81**	**127**
Image + Sensor	**0.94**	**0.90**	**0.92**	**0.91**	**50**

**Table 7:** Ablation study results: Impact of augmentation methods on disease detection model.

Augmentation Method	Precision	Recall	Accuracy	mAP@75
CutMix	**0.78**	**0.82**	**0.80**	**0.79**
Mosaic	**0.82**	**0.84**	**0.83**	**0.83**
GridMask	**0.86**	**0.81**	**0.84**	**0.83**
CutMix + Mosaic + GridMask	**0.94**	**0.90**	**0.92**	**0.91**

**Table 8:** Experimental results on extended dataset including fruit diseases.

Model	Precision	Recall	Accuracy	mAP@75	FPS
SSD	0.81	0.84	0.83	0.82	32
Retinadet	0.84	0.81	0.82	0.82	30
leafdetection	0.85	0.82	0.83	0.83	34
YOLOv8-E	0.87	0.83	0.85	0.84	38
DETR	0.89	0.84	0.86	0.85	41
Proposed Method	0.91	0.87	0.89	0.88	46

## Data Availability

Data are contained within the article.
